# Effect of acute acid-base disturbances on the phosphorylation of phospholipase C-γ1 and Erk1/2 in the renal proximal tubule

**DOI:** 10.14814/phy2.12280

**Published:** 2015-03-16

**Authors:** Lara A Skelton, Walter F Boron

**Affiliations:** Department of Physiology and Biophysics, Case Western Reserve University School of MedicineCleveland, Ohio, USA

**Keywords:** Acid-base, Erk1/2, phospholipase C-γ1, phosphothreonine, phosphotyrosine

## Abstract

The renal proximal tubule (PT) plays a major role in whole-body pH homeostasis by secreting H^+^ into the tubule lumen. Previous work demonstrated that PTs respond to basolateral changes in [CO_2_] and [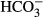] by appropriately altering H^+^ secretion—responses blocked by the ErbB inhibitor PD168393, or by eliminating signaling through AT_1_ angiotensin receptors. In the present study, we analyze phosphorylation of three downstream targets of both ErbBs and AT_1_: phospholipase C-γ1 (PLC-γ1), extracellular-regulated kinase 1 (Erk1), and Erk2. We expose rabbit PT suspensions for 5 and 20 min to our control (Ctrl) condition (5% CO_2_, 22 mmol/L 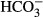, pH 7.40) or one of several conditions that mimic acid-base disturbances. We found that each disturbance produces characteristic phosphorylation patterns in the three enzymes. For example, respiratory acidosis (elevated [CO_2_], normal [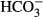]) at 20 min decreases PLC-γ1 phosphorylation at tyrosine-783 (relative to Ctrl). Metabolic acidosis (normal [CO_2_], decreased [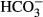]) for 5 min increases Erk1 phosphorylation (p-Erk1) but not p-Erk2, whereas metabolic alkalosis (normal [CO_2_], elevated [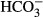]) for 5 min decreases p-Erk1 and p-Erk2. In the presence of CO_2_/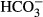, PD168393 blocks only two of eight induced decreases in phosphorylation. In two cases in which disturbances have no remarkable effects on phosphorylation, PD168393 unmasks decreases and in two others, increases. These drug effects provide insight into the roles of PD168393-sensitive kinases. Our results indicate that PLC-γ1.pY783, p-Erk1, and p-Erk2 in the PT change in characteristic ways in response to acute acid-base disturbances, and thus presumably contribute to the transduction of acid-base signals.

## Introduction^1^

The pH of the extracellular fluid (pH_o_) and the pH of intracellular fluid (pH_i_), which depends on pH_o_, are of profound importance because pH changes affect virtually every biological process (Roos and Boron 1982; Chesler 2003). Thus, it is not surprising that mammals closely regulate arterial pH. CO_2_ and 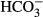 constitute the major pH buffer system. The body regulates pH_o_ to ∽7.40 by adjusting the ratio [CO_2_]_o_/[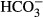]_o_, and regulates this ratio by independently controlling [CO_2_]_o_ and [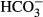]_o_. Controlling [CO_2_]_o_ is the respiratory system, which increases ventilation in response to elevated [CO_2_]_o_ or depressed pH_o_ (Boron 2012). Controlling [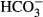]_o_ is the urinary system, which responds to acidosis by increasing its secretion of H^+^ into the urine, and moving a near-equivalent increment of HCO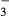 into the blood (Giebisch and Windhager 2009).

The renal proximal tubule (PT) is the major site of urinary H^+^ secretion, which proceeds as follows (Boron 2006): The PT cell secretes H^+^ into the lumen via two transporters on the apical membrane, the Na-H exchanger 3 (NHE3) (Biemesderfer et al. 1993; Wang et al. 2001) and the vacuolar-type H^+^-pump (Gluck et al. 1996). Typically, ∽98% of the secreted H^+^ titrates the HCO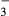 filtered by the glomeruli, with the remaining ∽2% titrating various other buffers in the tubule lumen [e.g., NH_3_, 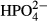, creatinine (Giebisch and Windhager 2009)]. Carbonic anhydrase IV (CA IV), GPI linked to the apical membrane, catalyzes the conversion of luminal 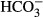 and the secreted H^+^ to H_2_O and CO_2_, which then enter the cell via AQP1 (Schnermann et al. 1998; Boron 2006). In the cytosol, CA II catalyzes the reconversion to H^+^ and 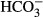, with the H^+^ recycling across the apical membrane into the lumen and the 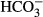 exiting across the basolateral membrane via the electrogenic Na^+^/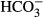 cotransporter NBCe1 (Boron and Boulpaep 1983; Romero et al. 1997).

The kidney can rapidly adjust its rate of H^+^ secretion, which—due to the titration of buffers other than 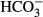 (see above)—is typically ∽2% higher than the rate at which 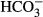 disappears from the lumen (i.e., 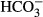 reabsorption, *J*hco_3_). For example, respiratory acidosis (RAc, a fall in blood pH caused by a rise in blood [CO_2_]) rapidly stimulates renal H^+^ secretion (Brazeau and Gilman 1953; Relman et al. 1953; Dorman et al. 1954). Eukaryotes have a variety of acid-base sensing mechanisms, many of which exist in the kidney (for reviews, see refs. Skelton et al. 2010; Tresguerres et al. 2010; Brown and Wagner 2012). However, none of these well-described systems appears to be operative in the acute sensing of acid-base disturbances by the PT (Skelton et al. 2010). Using out-of-equilibrium (OOE) CO_2_/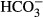 solutions (Zhao et al. 1995) to alter basolateral (BL; i.e., blood-side) [CO_2_], [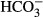], or pH one at a time with isolated perfused PTs—our laboratory made the surprising observation that *J*hco_3_ does not acutely respond to change in pH_BL_ or pH_i_ (Zhou et al. 2005). Rather, a rise in [CO_2_]_BL_ or a fall in [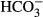]_BL_ causes *J*hco_3_ to rise, and the opposite changes cause *J*hco_3_ to fall. The potent and specific ErbB-family inhibitors PD168393 and BPIQ ablate the response to altering [CO_2_]_BL_ (Zhou et al. 2006a). Moreover, in PT suspensions, acid-base disturbances produce characteristic changes in the tyrosine phosphorylation of ErbB1 and ErbB2 (Skelton and Boron 2013). Thus, the question that arises is whether elements downstream of ErbB1/2 (Yarden and Sliwkowski 2001; Normanno et al. 2006; Roskoski 2014)—such as phospholipase C-γ1 (PLC-γ1), Erk1 (i.e., MAPK3 or p44), and Erk2 (i.e., MAPK1 or p42)—are part of the CO_2_/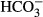 signaling cascade. Several lines of evidence (Zhou et al. 2006a, 2006b; Zhou and Boron 2008) indicate that the PT response to alterations in [CO_2_]_BL_ requires the luminal secretion of angiotensin II (ANG II) and its interaction with the apical angiotensin AT_1A_ receptor, which acts in part via PLCs, such as PLC-γ1 (Lea et al. 2002).

Others have implicated PLC-γ1 and Erk1/2 in the response to acid-base disturbances. The phosphoinositide-specific phospholipase C enzymes cleave the polar head group of phosphatidylinositol 4,5-bisphosphates (PIP_2_), generating the second messengers inositol 1,4,5-trisphosphate (IP_3_), which mobilizes Ca^2+^ from the smooth endoplasmic reticulum, and diacylglycerols (DAGs), which remain in the membrane and activate PKCs (Kamat and Carpenter 1997; Rebecchi and Pentyala 2000; Katan 2005; Bunney et al. 2012). Six distinct PLC families—β, γ, δ, and ζ, ε and η—are known. All possess X and Y motifs forming the catalytic core, and around this framework are assorted regulatory domains particular to each family. γ-family members PLC-γ1 and PLC-γ2 are distinguished by a string of SH2-SH2-SH3 domains; the SH2 domains enable recruitment to phosphotyrosine (pY) motifs, which are under the control of tyrosine kinases (Katan 2005) and phosphotyrosine phosphatases (Tiganis and Bennett 2007; Soulsby and Bennett 2009). For example, phosphorylation of PLC-γ1 on tyrosine residue 783 enables an intramolecular interaction between pY783 and the C-terminal SH2 domain, and is required—but not sufficient—for enzymatic activation (Kim et al. 1990; Sekiya et al. 2004; Poulin et al. 2005). PLC β, γ, and δ members are detected throughout the kidney and are important signaling mediators (Cha et al. 1998; Lea et al. 1998). PLC-γ1 is expressed in several nephron segments, including the PT, where it is detected diffusely in the cytoplasm (Cha et al. 1998; Lea et al. 1998). PLC-γ1 regulates NHE3 in cell culture models (Zachos et al. 2009). In LLC-PK1 cells, a PT model, ANG II binding to the AT_1_ receptor activates PLC-γ1, thereby leading to activation of calcineurin (Lea et al. 2002). PLC-γ1 also regulates phosphorylation of tight-junction proteins in MDCK cells (Ward et al. 2006).

Acute metabolic acidosis (MAc, a fall in blood pH caused by a fall in blood [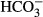]) activates Erk1/2 within 5 min in the OKP opossum PT cell line (Tsuganezawa et al. 2002). Rats gavaged with NH_4_Cl (to induce MAc) also exhibit elevated Erk1/2 activity within the renal cortex at 30 min. (Tsuganezawa et al. 2002). These results suggest a role for Erk1/2 in mediating acid-sensitive signaling.

In the present study, we examine the effect of acute acid-base disturbances on the phosphorylation status of PLC-γ1 and Erk1/2 in suspensions of rabbit PTs. The acid-base disturbances mimic the four classical disturbances—MAc, RAc, metabolic alkalosis (MAlk, a rise in blood pH caused by a rise in blood [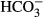]), and respiratory alkalosis (RAlk, a rise in blood pH caused by a fall in blood [CO_2_])—as well as fully compensated RAc (cRAc, a proportional rise in both blood [CO_2_] and [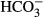] that holds pH constant). We examined the phosphorylation of PLC-γ1 at Y783, the phosphorylation of Erk1 at threonine 202 (T202) and Y204, and the phosphorylation of Erk2 at the two homologous residues T185 and Y187.

## Materials and Methods

### Media for PT suspension

For renal cortex dissection and PT enrichment, our base solution was half Dulbecco's modified Eagle's medium and half Ham's F-12 medium (DMEM-F12 50/50), purchased as a powder lacking l-glutamine and NaHCO_3_ (Catalog #90-091-PB, Corning, Cellgro® Mediatech Inc., Manassas, VA). The powder was diluted in Milli-Q water and we lowered the temperature to 0°C in an ice bath. We supplemented this DMEM-F12 50/50 media with 15 mmol/L HEPES, 2 mmol/L l-glutamine, 2 mmol/L heptanoic acid (Catalog #75190; Sigma-Aldrich, St. Louis, MO), and 2 mmol/L l-Lactic acid (30% in water by weight, Sigma-Aldrich, Catalog #L1875), and then titrated to pH 7.40. We added 15 mmol/L NaHCO_3_ and gassed with 3% CO_2_/balance air. We adjusted the osmolality to 310 ± 5 mOsm with NaCl or H_2_O. We call this final solution DMEM-F12.

### Tissue procurement and proximal-tubule preparation

We used kidneys from Female New Zealand White rabbits, housed and handled according to protocols approved by IACUC at Case Western Reserve University. Under anesthesia (∽10 mL intravenously administered 5% pentobarbital sodium in H_2_O) rabbits were euthanized by exsanguination. The kidneys were removed, decapsulated, and sliced into 5–6 mm coronal slices in ice-cold DMEM-F12 prepared as above. Proximal-tubule suspensions were prepared using a method adapted from Doctor et al. (1998). The renal cortex of each coronal slice was trimmed and finely diced before digestion in 0.5 mg/mL collagenase type IV (Catalog #C5138, Sigma-Aldrich) in DMEM-F12, shaken at 225 rpm for 10 min at 37°C. The digested cortex was collected by centrifugation at 150 *g* for 1 min and washed three times with DMEM-F12 to remove residual collagenase.

The digested renal cortex was placed in DMEM-F12 on ice and the tubules separated using a flat glass pestle followed by trituration through a 10-mL pipette. The cortical tubules in suspension were washed three times in DMEM-F12, harvesting each time at 150 *g* for 2 min, divided into four equal aliquots and gently shaken at 4°C for 1 h in freshly gassed DMEM-F12 media. The cortical tubules were resuspended into four 25-mL aliquots of ice-cold 45% Percoll solution containing: 45 mL Percoll, 50 mL freshly gassed DMEM-F12, 4.5 mL 10× phosphate-buffered saline (PBS) from PBS tablets without calcium or magnesium (Catalog #2810305, MP Biomedicals LLC, Solon, OH). The tubules were then separated on a self-forming gradient by centrifugation at 25 000 *g* for 35 min at 4°C. The tubules migrated into strata, the bottom, closely-migrating bands F4/F5 were enriched for PTs as determined by western blotting analysis (Skelton and Boron 2013), and were harvested for experimentation.

### Subjection of PTs to acid-base disturbances

The compositions of our physiological solutions are shown in Table1. We generated gas mixtures indicated for the CO_2_-containing solutions (% of CO_2_, 21% O_2_, and balance N_2_) using a computer-controlled gas-mixing system (Series 4000; Environics, Tolland, CT), and bubbling the solutions for 30–40 min. The solutions for which CO_2_ is not specified in Table1 (labeled nominally CO_2_-free solutions) were equilibrated with room air. We purchased butyric acid, 99+ % from Sigma-Aldrich (B103500); lyophilized purified mouse epidermal growth factor EGF1 from AbD Serotec (AbD Serotec, MorphoSys, Martinsried/Planegg, Germany), and PD168393 from Calbiochem (Catalog #513033, Calbiochem, La Jolla, CA).

**Table 1:** The compositions of physiological solutions^1^

Solution name	Nominally CO_2_/ 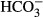 -free HEPES ± PDI68393	Nominally CO_2_/ 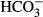 -free HEPES + 20 mmol/L butyrate pH 7.4	Nominally CO_2_/ 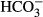 -free HEPES + 20 mmol/L butyrate pH 7.1	2.5% CO_2_ 22 mmol/L 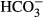 ± PD168393	5% CO_2_ 11 mmol/L 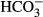 ± PD168393	5% CO_2_ 22 mmol/L 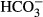 ± PD168393	5% CO_2_ 44 mmol/L 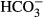 ± PD168393	10% CO_2_ 22 mmol/L 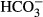 ± PD168393	10% CO_2_ 44 mmol/L 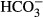 ± PD168393
Solutes	HEPES	Butyrate/pH 7.4	Butyrate/pH 7.1	RAlk	MAc	Ctrl	MAlk	RAc	cRAc
CO_2_	0	0	0	0.6	1.2	1.2	1.2	2.4	2.4
NaHCO_3_	0	0	0	22	11	22	44	22	44
NaCl	133	115	115	111	123	111	89	111	89
KCl	5	5	5	5	5	5	5	5	5
H_3_PO_4_	0.2	0.2	0.2	0.2	0.2	0.2	0.2	0.2	0.2
CaCl_2_	1	1	1	1	1	1	1	1	1
MgSO_4_	1.2	1.2	1.2	1.2	1.2	1.2	1.2	1.2	1.2
Glucose	10.5	10.5	10.5	10.5	10.5	10.5	10.5	10.5	10.5
l-Lactic acid	2	2	2	2	2	2	2	2	2
l-Glutamine	2	2	2	2	2	2	2	2	2
HEPES	15	15	15	15	15	15	15	15	15
n-Butyric acid (i.e., butyrate)	0	20	20	0	0	0	0	0	0

1 In some experiments, we added PD168393 (50 nmol/L) to solutions. In others, we added EGF (1 ng/mL) to the HEPES solution.

We divided the enriched PT suspension into ∽5 mL aliquots in 50-mL, conical-bottom polypropylene tubes with screw caps (Catalog #352070; BD Biosciences, Durham, NC) and diluted the tubule suspension with freshly gassed DMEM-F12 to ∽10 mL total volume. The PTs were allowed to rest at room temperature for 30 min prior to experimentation. Each aliquot was gently pelleted at 150 g for 1 min, the DMEM-F12 was decanted, and the PTs were resuspended in one of the prewarmed physiological solutions (Table1) and incubated for 5 min or for 20 min at 37°C. The PTs were then harvested by centrifugation at 1000 *g* for 30 sec, the physiological solutions were decanted, the pelleted PTs were immediately flash frozen in liquid nitrogen and then stored at −80°C until processing for western-blot analysis.

### Protein extraction and western blotting

Frozen PT pellets were rapidly thawed, placed on ice, and lysed by trituration through a 21-gauge needle in a buffer containing: 25 mmol/L nominally CO_2_/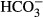-free HEPES, pH 7.50, 100 mmol/L NaCl, 50 mmol/L NaF, 10 mmol/L Na-pyrophosphate (Na_4_P_2_O_7_), 1 mmol/L EDTA, and 1% Nonidet P-40 (AB01425; American Bioanalytical, Natick, MA) to which we added protease cocktail inhibitor cocktail (P8340; Sigma-Aldrich) at 1:25 (v/v). After lysis, we added 2 mmol/L Na-orthovanadate (Na_3_VO_4_). The total protein content was determined using a BCA protein assay (Catalog #23227, Thermo Scientific Pierce). Equal amounts of protein were loaded onto NuPAGE Novex 4–12% Bis Tris gels (NP0322 and NP0366, Invitrogen), and resolved by electrophoresis. The proteins were electrophoretically transferred to PVDF membrane and blocked in TBST (Tris-buffered saline plus Tween: 10 mmol/L Tris-HCl, pH 7.50, 150 mmol/L NaCl, 0.01% Tween 20), supplemented with 5% milk powder, 2 mmol/L Na_3_VO_4_, at room temperature for 1 h. The membranes were probed with primary antibodies overnight at 4°C, after which they were washed to remove unbound antibodies, incubated with secondary antibodies conjugated to horseradish peroxidase, and then washed. We detected immunoreactive bands with ECL2 reagent (Catalog # 80196; Thermo Scientific Pierce, Rockford, IL) using X-ray film. Before reprobing, blots were stripped well by incubation in Restore™ Plus western-blot stripping buffer (Catalog #46430; Thermo Scientific) for 30 min at 37°C with agitation.

We used the following primary antibodies for detection of PLC-γ1 and Erk1/2 by western blot: (1) a rabbit polyclonal antibody directed against the phosphorylated PLC-γ1.pY783 (#2821, Cell Signaling Technology, Danvers, MA), (2) a mouse monoclonal directed against PLC-γ1 (sc-7290, Santa Cruz Biotechnology, Inc. Santa Cruz, CA), (3) a rabbit polyclonal directed against phospho-Erk1/2 (#9101, Cell Signaling Technology) at the conserved sites T202/Y204 in Erk1 (i.e., MAPK3 or p44) and T185/Y187 in Erk2 (i.e., MAPK1 or p42), and (4) a rabbit polyclonal directed against Erk1/2 (#AB3053; EMD Millipore Billerica, MA).

Secondary antibodies used were a goat affinity-purified antibody to rabbit IgG, HRP conjugated (AP132P, Millipore), and goat affinity purified antibody to mouse IgG, HRP conjugated (#55563; MP Biomedicals, LLC).

### Image acquisition

We scanned films as 16-bit grayscale images using an Epson Perfection V500 photo scanner, on positive film setting at high resolution (600–800 dpi) and performed densitometry analyses using ImageJ software (NIH). For analysis of PLC-γ1, we normalized the major pY783 band (migrating at ∽150 kDa) to the corresponding band reprobed total PLC-γ1. We normalized p-Erk1 to reprobed total Erk1 (p44 migrating at ∽44 KDa), and normalized p-Erk2 to total Erk2 (p42 migrating at ∽42 KDa).

### Data analysis

Following densitometry and normalization of phospho-specific signals to reprobed total-detected protein (i.e., PLC-γ1, Erk1, or Erk2), as described above, we normalized to the value obtained from our nominally CO_2_/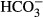-free HEPES solution in Figures 3A, 4A, 7A, 8A, or to our control 5% CO_2_/22 mmol/L 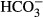 (Ctrl) treatment in Figures 3B, 4B, 7B, 8B. For the first of our two analyses of experiments with PD168393, we compare the drug-treated tubules with the subset of drug-free rabbit PTs examined alongside the PD168393-treated tubules. In Figures 3C, 4C, 7C, 8C, we normalized the data for both PD168393-treated and drug-free PTs to our Ctrl treatment (in the absence of drug), and then divided the normalized data from PD168393-treated PTs by the normalized drug-free data for that particular acid-base condition. In Figures 3D, 4D, 7D, 8D, we divided the normalized the data from PD168393-treated PTs for a particular acid-base condition by the normalized the data from PD168393-treated PTs incubated under Ctrl conditions. In statistical analyses of normalized western-blot data, our final step was to perform a log_2_ transformation of each data point, thereby permitting unbiased comparisons of increases vs. decreases in ratio. As described by others (Quackenbush 2002), we discarded the ∽5% of data points in which log_2_ was more than 2× standard deviations from the mean of the set. We used Excel 2010 (Microsoft Corporation, Redmond, WA) to perform statistical analyses, with rounded *P* < 0.05 being considered significant.

## Results

### General approach

In the experiments that follow, we transferred aliquots of PT suspensions (prepared from each of 12 rabbits) from DMEM medium to as few as nine, or as many as 13, treatment solutions that we selected from a pool of 17 total such solutions: (1) four nominally CO_2_/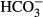-free solutions without inhibitor, (2) six CO_2_/HCO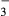-containing solutions without inhibitor, (3) one nominally CO_2_/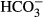-free solution with the ErbB inhibitor PD168393, and (4) six CO_2_/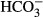-containing solutions with PD168393. Table1 provides the compositions of the inhibitor-free solutions. After a 5- or 20-min incubation in the treatment solution, we flash froze the PT suspension to prevent further kinase/phosphatase action. We later extracted protein from PT preparations, resolved by SDS-PAGE, obtained western blots, and then probed first with a phosphorylation-state–dependent antibody (anti-PLC-γ1.pY783 or anti-phospho-Erk1/2), stripped the blot, and then reprobed with a phosphorylation-state–independent antibody (either anti-PLC-γ1.total or anti-Erk1/2.total).

### Western blots of PLC-γ1/pY783

**Figure 1:**
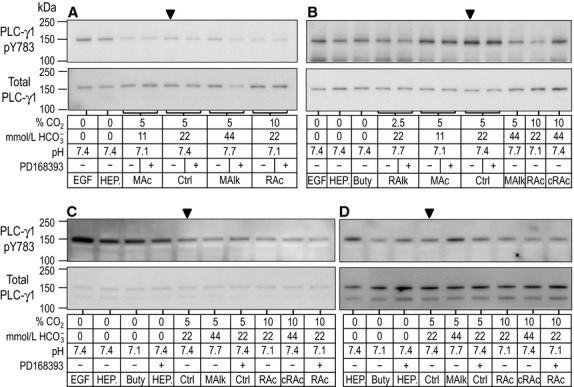
Sample western blots of PLC-γ1.pY783 immunoreactivity after 5-min treatments. Rabbit PTs were challenged for 5 min with one of several nominally CO_2_/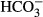-free HEPES solutions or CO_2_/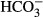-containing solutions mimicking acid-base disturbances. Experiments were performed as a series of overlapping assays with representative blots shown in panels A–D. For each lane, the upper part shows the immunoreactivity for PLC-γ1.pY783. Beneath that, is the corresponding reprobe for total PLC-γ1. At the bottom is a tabular summary of treatment conditions. [We have made every effort to select blots that are representative of the averaged data but, due to the sample-sample variability associated with working with native tissue, not every band on each selected blot conforms to the average. The sample variability is faithfully described by the error bars and statistical analysis in the bar charts that accompany the analysis of these blots (e.g. Fig. 2 reports the variability of the sample data in this figure).]

**Figure 2:**
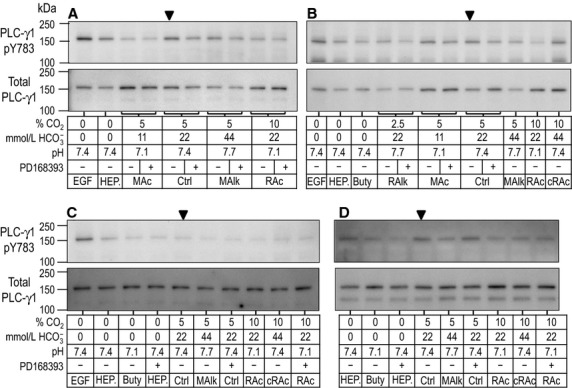
Sample western blots of PLC-γ1.pY783 immunoreactivity after 20-min treatments. The layout of this figure is identical that of Figure1, with the exception of the duration of exposure of PTs to treatment solutions.

In Figure1A–D we show example western blots obtained after 5-min treatments, representing experiments from four rabbits (one rabbit in each of panels A, B, C, and D). Similarly, Figure2A–D shows four example blots obtained after 20-min treatments. PLC-γ1.pY783 appears predominantly as a band at ∽150 kDa (upper portion of each panel), the predicted MW. Reprobing for PLC-γ1.total (lower portion of each panel), we detect a corresponding major band at the same MW, and sometimes a fainter ∽120-kDa band. For densitometry analysis, we consider only the ∽150-kDa species of PLC-γ1. The lanes for the Ctrl condition (5% CO_2_/22 mmol/L HCO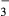) without drug are marked with arrowheads. For example, we see in Figure1A–C that EGF in nominally CO_2_/HCO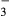-free HEPES appears to modestly enhance the PLC-γ1.pY783 signal (EGF vs. nominally CO_2_/HCO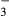-free HEPES). In the next two sections, we summarize the effects of 5-min and 20-min exposures to our treatment solutions on the phosphorylation state of PLC-γ1.

### The response of PLC-γ1/pY783 to 5-min acid-base treatments

**Figure 3:**
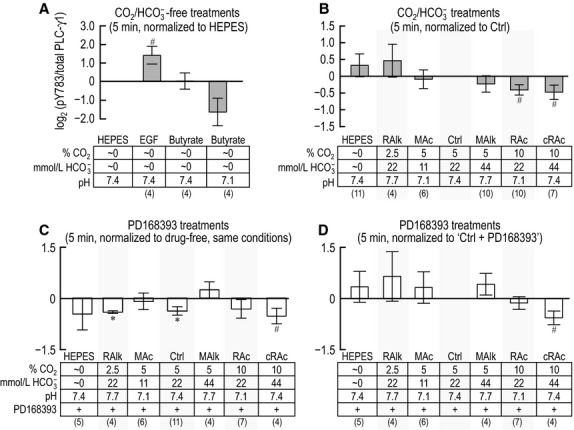
Quantification of PLC-γ1 immunoreactivity at pY783 after 5-min exposure of PTs to treatment solutions. We show densitometry data from western blots, like those in Figure1. For each treatment, we first normalize PLC-γ1.pY783 to total PLC-γ1 immunoreactivity in the same lane (reprobed blot). (A) PLC-γ1.pY783/(total PLC-γ1) for nominally CO_2_/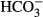-free treatment solutions, each normalized to PLC-γ1.pY783/(total PLC-γ1) for the nominally CO_2_/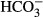-free solution (HEPES) on the same blot. (B) PLC-γ1.pY783/(total PLC-γ1) for HEPES and CO_2_/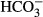 treatment solutions, each normalized to PLC-γ1.pY783/(total PLC-γ1) for the Ctrl treatment (5% CO_2_/22 mmol/L 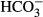) on the same blot. (C) PLC-γ1.pY783/(total PLC-γ1) for treatments like those in panel B, but with and without the ErbB inhibitor PD168393, each normalized to PLC-γ1.pY783/(total PLC-γ1) for the equivalent treatment without PD168393 on the same blot. (D) PLC-γ1.pY783/(total PLC-γ1) for PD168393-treated PTs, as in panel C, but instead normalized to PLC-γ1.pY783/(total PLC-γ1) for PD168393-treated PTs incubated in the Ctrl solution (5% CO_2_/22 mmol/L 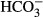) on the same blot. Sample size for each bar is shown in parentheses beneath the legend. ^#^Denotes a bar for which *P* < 0.05 (compared to zero, one-tailed, paired *t*-test); in the text, we state that the parameter “tends” to rise/fall. *Denotes a bar that is significantly different from zero, even after the very conservative Bonferroni correction (*P* < 0.05 divided by the number of treatment solutions considered in each panel); in the text, we refer to the change as “significant.”

Figure3 summarizes data for 11 experiments like those in Figure1. In each of the four panels of Figure3, the ordinate is the ratio of the PLC-γ1.pY783 signal (e.g., Fig.1A, upper portion) to total PLC-γ1 immunoreactivity (e.g., Fig.1A, lower portion), normalized to a standard condition that is unique for each panel. Figure3A summarizes (PLC-γ1.pY783)/(PLC-γ1.total) ratios for PTs that we subjected to 5-min incubations in our four nominally CO_2_/HCO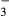-free treatment solutions. In this panel, we normalize the ratios, for each PT preparation, to the ratio that we calculate for nominally CO_2_/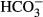-free HEPES-treated PTs, and then perform a log_2_ transformation. After a 5-min incubation with EGF, PLC-γ1.pY783 tends to increase, as defined in the legend for Figure3. Butyrate is expected to cause a fall in intracellular pH (due to the influx of butyric acid) that is at least transient; the pH_o_ decrease from 7.4 to 7.1 has the added effect of increasing [butyric acid]_o_. Here, butyrate/pH 7.1 barely fails to reach the 0.05 level of significance in a paired *t*-test.

Figure3B summarizes (PLC-γ1.pY783)/(PLC-γ1.total) ratios for PTs subjected to 5-min incubations in nominally CO_2_/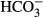-free HEPES or in one of our six drug-free CO_2_/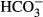-containing treatment solutions. In this panel, we normalize the ratios for each of the seven conditions to the ratio that we calculate for PTs exposed to our Ctrl solution (5% CO_2_/22 mmol/L 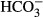/pH 7.40). We see a tendency for pY783 to decrease when [CO_2_] is high (Fig.3B, RAc or cRAc vs. Ctrl).

In parallel with the treatments that generated the data shown in Figure3B, we also treated PT aliquots from some rabbits with solutions that included the ErbB-family blocker PD168393. We summarize these data in Figure3C, which shows, for each treatment, the log_2_ transformation of the (PLC-γ1.pY783)/(PLC-γ1.total) ratio with PD168393, normalized to the corresponding ratio without drug. PD168393 produces a significant decrease in pY783 under the RAlk and Ctrl conditions, and a tendency to decrease with cRAc. These results are consistent with the hypothesis that ErbB-dependent kinase activity contributes to the baseline PLC-γ1.pY783 phosphorylation state under RAlk and Ctrl conditions at 5 min, with a tendency to contribute with cRAc.

The lack of PD168393-sensitivity after 5 min treatment of PTs in HEPES, MAc, MAlk, and RAc (Fig.3C) is consistent with the hypothesis that the phosphorylation state of PLC-γ1.pY783 during these treatments does not depend upon the action of an ErbB-family kinase.

In Figure3D, we reanalyze the PD168393 data by normalizing each log_2_ transformation to the ratio for the same PTs exposed to our Ctrl solution in the presence of PD168393. Thus, Figure3D is analogous to Figure3B but, throughout, reflects the presence of the ErbB inhibitor. We observe two patterns: (1) As in Figure3B, we see in Figure3D a tendency for cRAc to decrease PLC-γ1.pY783. Thus, PD168393-sensitive kinases are not necessary for cRAc to lower pY783—even though PD168393 reduces pY783 to varying degrees under both Ctrl and cRAc conditions. (2) On the other hand, whereas RAc has a tendency to decrease pY783 in Figure3B, it does not in Figure3D, consistent with the hypothesis that PD168393-sensitive kinases are necessary for RAc to lower pY783.

### The response of PLC-γ1/pY783 to 20-min acid-base treatments

**Figure 4:**
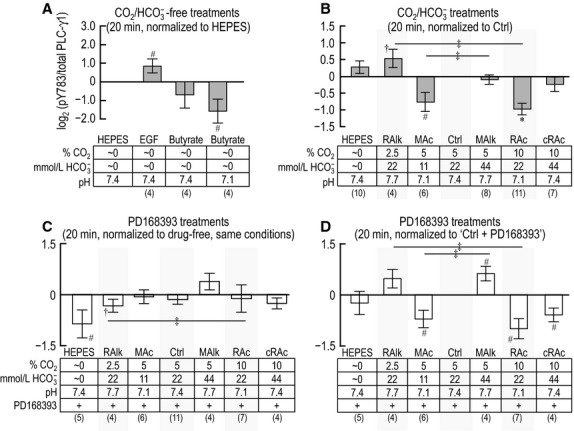
Quantification of PLC-γ1 immunoreactivity at pY783 after 20-min exposure of PTs to treatment solutions. This figure is identical to Figure3, with the exception of the duration of exposure of PTs to treatment solutions. Sample size for each bar is shown in parentheses beneath the legend. †Denotes a bar for which all constituent data points are >0 (or <0) but not statistically different from 0 (one-tailed, paired t-test). ^#^Denotes a bar for which *P* < 0.05 (compared to 0, one-tailed, paired *t*-test); in the text, we state that the parameter “tends” to rise/fall. *Denotes a bar that is significantly different from 0, even after the very conservative Bonferroni correction (*P* < 0.05 divided by the number of treatment solutions considered in each panel); in the text, we refer to the change as “significant.” ^‡^Denotes a pair of bars that differ significantly from one another in a one-tailed paired *t*-test; in the text, we state that the parameters “tend” to differ.

Figure4 summarizes data for 11 experiments like those in Figure2. The four panels in Figure4 are analogous to their counterparts in Figure3, except that here in Figure4, the exposure is for 20 min rather than 5 min. For experiments conducted in the absence of CO_2_/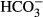, Figure4A reveals at 20 min a pattern that is similar to that in Figure3A. After a 20-min incubation with EGF, PLC-γ1.pY783 tends to increase. However, now butyrate at pH 7.1 exhibits a tendency to decrease pY783.

The data in Figure4B summarizes (PLC-γ1.pY783)/(PLC-γ1.total) ratios for PTs subjected to 20-min incubations in nominally CO_2_/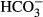-free HEPES or in one of our six CO_2_/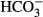-containing treatment solutions, all normalized to the (PLC-γ1.pY783)/(PLC-γ1.total) ratio at 20 min in Ctrl. Compared to the analogous 5-min data, five differences are noteworthy: (1) RAlk at 20 min now consistently increases the ratio (PLC-γ1.pY783)/(PLC-γ1.total), (2) RAc now significantly decreases pY783, and (3) cRAc now shows no effect on pY783. Also different from the situation at 5 min, (4) MAc at 20 min exhibits a tendency to decrease pY783. Moreover, (5) each acidosis (RAc and MAc) tends to differ from its corresponding alkalosis (RAlk and MAlk).

Unlike the situation at 5 min, the summary of PD168393 data in Figure4C shows that Ctrl and cRAc no longer produce any indication of a decrease in the (PLC-γ1.pY783)/(PLC-γ1.total) ratio in PD168393 normalized to the ratio in the corresponding condition in the absence of drug. Moreover, RAlk produces only a consistent decrease in the signal. On the other hand, HEPES at 20 min now produces a tendency for the signal to decrease.

Figure4D, a reanalysis of the PD168393 data—but normalized to Ctrl in the presence of drug—reveals three patterns. (1) Similar to the pattern in Figure4B, the two acidoses (MAc and RAc) in Figure4D have a tendency to lower pY783—a pattern reminiscent of that for cRAc at 5 min in Figure3B/D. Thus, the action of PD168393-sensitive kinases are not necessary for the acidosis-induced decreases in pY783. (2) Although MAlk lacks a noteworthy effect in Figure4B (–PD168393), it produces in Figure4D (+PD168393) a tendency for pY783 to rise. One possibility^2^ is that, at 20 min in the absence of drug, MAlk inhibits PD168393-sensitive kinases and thereby lowers pY783, but that MAlk simultaneously stimulates other kinases and thereby raises pY783. The net effect would be no change in pY783 in the absence of drug (Fig.4B). However, in the presence of the drug, MAlk would raise pY levels via these other kinases. (3) Although cRAc is not noteworthy in Figure4B, it produces a tendency for pY783 to fall in Figure4D (+PD168393). One possibility is that, at 20 min in the absence of drug, cRAc tends to raise pY783 via PD168393-sensitive kinases, but to lower pY783 via other kinases. Thus, in the presence of the drug, cRAc would lower pY levels via these other kinases.

### Western blots of phospho-Erk1/2

**Figure 5:**
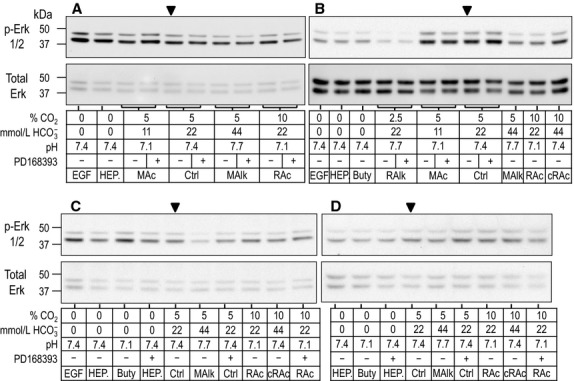
Sample western blots of p-Erk1/2 immunoreactivity after 5-min treatments. Rabbit PTs were challenged for 5 min with one of several nominally CO_2_/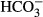-free HEPES solutions or CO_2_/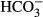-containing solutions mimicking acid base disturbances. Experiments were performed as a series of overlapping assays with representative blots shown in A–D. For each lane, the upper part shows the immunoreactivity for p-Erk1 (p44, upper band) and p-Erk2 (p42, lower band). Beneath that, is the corresponding reprobe for total Erk1/2. At the bottom is a tabular summary of treatment conditions. The antibody that we used recognizes human pT202/pY204 in p-Erk1 and pT185/pY187 in p-Erk2.

**Figure 6:**
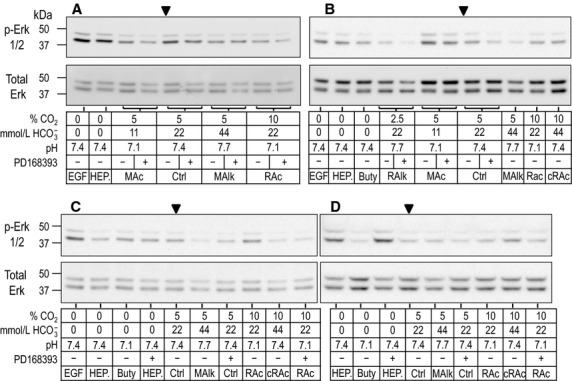
Sample western blots of p-Erk1/2 immunoreactivity after 20-min treatments. The layout of this figure is identical that of Figure5, with the exception of the duration of exposure of PTs to treatment solutions.

Figure5 shows western blots obtained after 5-min treatments, representing experiments from four rabbits (1 rabbit in each panel), and Figure6 shows comparable blots obtained after 20-min treatments. As expected, we detect two bands by probing with either the phospho-specific Erk1/2 (p-Erk1/2, upper portion of each panel) or Erk1/2.total, one migrating at ∽44 kDa (Erk1) and the other at ∽42 kDa (Erk2). The Ctrl lane without drug is marked with an arrowhead in each set of panels. In the next section, we consider the effects of a 5-min exposure to our treatment solutions on the phosphorylation state of Erk1 and Erk2, and in the section after that, the effect of 20-min treatments.

### Response of p-Erk1/2 to 5-min acid-base disturbances

**Figure 7:**
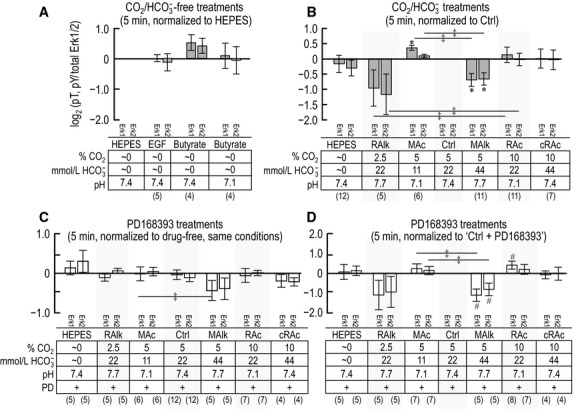
Quantification of p-Erk1/2 immunoreactivity—pT202/pY204 for Erk1 and pT185/pY187 for Erk2—after solution treatments of 5 min. We show densitometry data from western blots, like those in Figure5. For each treatment, we first normalize p-Erk1 and p-Erk2 immunoreactivity to total Erk1 or Erk2 immunoreactivity—that is, (p-Erk1)/(Erk1.total) and (p-Erk2)/(Erk2.total)—in the same lane (reprobed blot). (A) (p-Erk1)/(Erk1.total) and (p-Erk2)/(Erk2.total) for nominally CO_2_/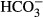-free treatment solutions, each normalized to (p-Erk1)/(Erk1.total) or (p-Erk2)/(Erk2.total) for the nominally CO_2_/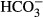-free solution (HEPES) on the same blot. (B) (p-Erk1)/(Erk1.total) and (p-Erk2)/(Erk2.total) for HEPES and CO_2_/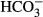 treatment solutions, each normalized to (p-Erk1)/(Erk1.total) or (p-Erk2)/(Erk2.total) for the Ctrl treatment (5% CO_2_/22 mmol/L 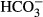) on the same blot. (C) (p-Erk1)/(Erk1.total) and (p-Erk2)/(Erk2.total) for treatments like those in panel B, but with the ErbB inhibitor PD168393, each normalized (p-Erk1)/(Erk1.total) or (p-Erk2)/(Erk2.total) for the equivalent treatment without PD168393 on the same blot. (D) (p-Erk1)/(Erk1.total) and (p-Erk2)/(Erk2.total) for PD168393-treated PTs, as in panel C, but instead normalized to (p-Erk1)/(Erk1.total) or (p-Erk2)/(Erk2.total) for PD168393-treated PTs incubated in the Ctrl solution (5% CO_2_/22 mmol/L 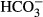) on the same blot. Sample size for each bar is shown beneath the legend for the panel. PD, PD168393. ^#^Denotes a bar for which *P* < 0.05 (compared to zero, one-tailed, paired t-test); in the text, we state that the parameter “tends” to rise/fall. *Denotes a bar that is significantly different from zero, even after the very conservative Bonferroni correction (*P* < 0.05 divided by the number of treatment solutions considered in each panel); in the text, we refer to the change as “significant.” ^‡^Denotes a pair of bars that differ significantly from one another in a one-tailed paired *t*-test; in the text, we state that the parameters “tend” to differ.

In Figure7A, the paired bars summarize the (p-Erk1)/(Erk1.total) and (p-Erk2)/(Erk2.total) ratios for PTs incubated for 5 min in our four nominally CO_2_/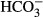-free treatment solutions. For each PT preparation, we normalized each ratio to the (p-Erk1)/(Erk1.total) or (p-Erk2)/(Erk2.total) ratio that we calculated for nominally CO_2_/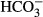-free HEPES-treated PTs (Fig.7A). None of these 5-min treatments—even the treatment with EGF—cause a noteworthy change in Erk1 or Erk2 phosphorylation.

**Figure 8:**
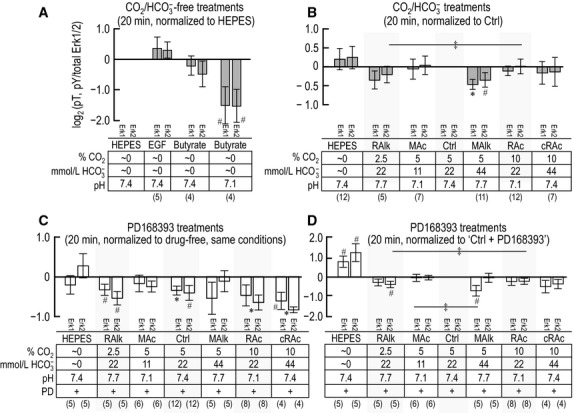
Quantification of p-Erk1/2 immunoreactivity—pT202/pY204 for Erk1 and pT185/pY187 for Erk2—after solution treatments of 20 min. This figure is identical to Figure7, with the exception of the duration of exposure of PTs to treatment solutions. Sample size for each bar is shown in parentheses beneath the legend. ^#^Denotes a bar for which *P* < 0.05 (compared to 0, one-tailed, paired *t*-test); in the text, we state that the parameter “tends” to rise/fall. PD, PD168393. *Denotes a bar that is significantly different from 0, even after the very conservative Bonferroni correction (*P* < 0.05 divided by the number of treatment solutions considered in each panel); in the text, we refer to the change as “significant.” ^‡^Denotes a pair of bars that differ significantly from one another in a one-tailed paired *t*-test; in the text, we state that the parameters “tend” to differ.

Figure7B summarizes the (p-Erk1)/(Erk1.total) and (p-Erk2)/(Erk2.total) ratios for PTs subjected to a 5-min incubation in nominally CO_2_/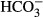-free HEPES plus one of our six drug-free CO_2_/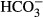-containing treatment solutions. Here, we normalized each ratio to the ratio that we calculate for PTs exposed to our Ctrl solution. Four results are noteworthy: (1) MAc significantly enhances phosphorylation of Erk1 (but not Erk2) vs. Ctrl in a 5-min exposure. This is the only example in the present study^3^ of an increase in phosphorylation caused by an acid-base disturbance in the absence of PD168393. In addition, MAlk significantly reduces both (2) p-Erk1 and (3) p-Erk2 vs. Ctrl. Finally, (4) compared to their acidotic counterparts (MAc and RAc), the alkalotic treatments (MAlk and RAlk) each tend to lower both p-Erk1 and p-Erk2.

In parallel with the treatments that generated the data shown in Figure7B, we also treated PT aliquots from some rabbits with solutions that included PD168393. Figure7C summarizes this subset of data, expressed as the log_2_ transformation of the (p-Erk1)/(Erk1.total) and (p-Erk2)/(Erk2.total) ratios with PD168393, normalized to the comparable ratio in the absence of drug. Figure7C suggests that the drug does not have a significant effect on p-Erk1/2 for any one of the treatments, although PD168393 does have a tendency to reduce p-Erk-1 more during MAlk than MAc.

In Figure7D, we reanalyze the PD168393 data—but normalize to Ctrl in the presence of drug. Two patterns emerge in the presence of PD168393 compared to the absence of the drug in Figure7B: (1) Similar to the pattern in Figure7B, MAlk in Figure7D has a tendency to lower p-Erk1 and p-Erk2. Thus, PD168393-sensitive kinases are not necessary for the MAlk-induced decreases in p-Erk1/2—reminiscent of the pattern that we noted for cRAc at 5 min in Figure3B/D, and for MAc and RAc at 20 min in Figure4B/D. (2) Although MAc increases p-Erk1 in Figure7B (–PD168393), MAc has no noteworthy effect in Figure7D (+PD168393). One possible explanation these Figure7B/D data is that MAc leads to the stimulation of a PD168393-sensitive kinase or the inhibition of a phosphatase that is activated by a PD168393-sensitive kinase. (3) Although RAc in Figure7B has no effect on p-Erk1, RAc in Figure7D has a tendency to increase p-Erk1—reminiscent of the pattern for MAlk on PLC-γ1.pY783 at 20 min in Figure4.

### Response of p-Erk1/2 to 20-min acid-base disturbances

Figure8A shows an analysis similar to that in Figure7A, but for treatments of 20 min. Note that even a 20-min treatment with EGF does not cause a significant elevation of Erk1/2 phosphorylation (Fig.8A, HEPES vs. EGF). However, different from our observation at 5 min, we see a tendency at 20 min for Butyrate, pH 7.10 to decrease the phosphorylation of both Erk1 and Erk2 (Fig.8A, HEPES vs. Butyrate, pH 7.10).

Figure8B is similar to Figure7B, but summarizes data for PTs treated for 20 min. As was the case for the 5-min exposure, MAlk decreases p-Erk1, but now produces only a tendency for p-Erk2 to decrease. Also unlike the situation after 5 min, MAc no longer increases p-Erk1. Moreover, the only comparison between sets of acid-base disturbances that remains noteworthy here at 20 min is the tendency for p-Erk2 to be lower in RAlk than RAc.

Figure8C is similar to Figure7C, but summarizes the effect of PD168393 at 20 min. Unlike the situation at 5 min, where the drug had no noteworthy individual effects on p-Erk1/2, at 20 min, PD168393 significantly reduces phosphorylation in three cases: p-Erk1 under Ctrl conditions, p-Erk2 with RAc, and p-Erk2 with cRAc. Moreover, PD168393 treatment at 20 min produces a tendency for p-Erk1/2 to fall with RAlk, p-Erk2 to fall with Ctrl, and p-Erk1 to fall with cRAc.

The reanalysis of the PD168393 data in Figure8D reveals three noteworthy effects. (1) Although RAlk has no effect in Figure8B, it tends to lower p-Erk2 in Figure8D—a pattern reminiscent of that for cRAc on PLC-γ1.pY783 at 20 min in Figure4. (2) MAlk lowers p-Erk1 in Figure8B and also tends to lower p-Erk1 in Figure8D—a pattern reminiscent of those for MAlk on p-Erk1/2 at 5 min in Figure7, cRAc on pY783 at 5 min in Figure3, and for MAc and RAc on pY783 at 20 min in Figure4. Thus, PD168393-sensitive kinases are not necessary for MAlk to lower p-Erk1. (3) Whereas MAlk tends to lower p-Erk2 in Figure8B, it has no effect in Figure8D—a pattern reminiscent of that for RAc on PLC-γ1.pY783 at 5 min in Figure3. Thus, PD168393-sensitive kinases are necessary for MAlk to lower p-Erk2 at 20 min.

### Comparison of Ctrl data at 5 and 20 min

**Figure 9:**
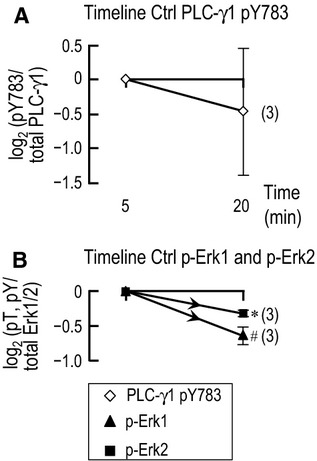
Comparison of PLC-γ1 and Erk1/2 phosphorylation following Ctrl treatment for 5 and 20 min. Tubule material from the Ctrl treatment (5% CO_2_/22 mmol/L 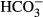) for 5 or 20 min from the same PT preparations were run side-by side on the same western blot and analyzed. PLC-γ1.pY783, p-Erk1, or p-Erk2 immunoreactivities were normalized to the corresponding total-protein immunoreactivities on reprobed blots. The 5-min and 20-min values were then normalized to the 5-min value, log_2_ transformed, and plotted as a function of time for (A) PLC-γ1.pY783 or (B), p-Erk1 and p-Erk2. ^#^Denotes data for which *P* < 0.05 (compared to zero, one-tailed, paired *t*-test); in the text, we state that the parameter “tends” to rise/fall. *Denotes data that are significantly different from zero, even after the very conservative Bonferroni correction (*P* < 0.05 divided by the number of treatment solutions considered in each panel); in the text, we refer to the change as “significant.”

In the B panels of Figures3, 4, 7, 8, we normalized data obtained with various acid-base disturbances to Ctrl data (5% CO_2_/22 mmol/L 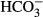) obtained at the same time (i.e., 5 or 20 min). In order to determine whether the phosphorylation status of PLC-γ1, Erk1, or Erk2 changed with time under Ctrl conditions, we performed the western blot analysis summarized in Figure9. After the 30-min incubation in DMEM-F12 medium (3% CO_2_), we resuspended the tubules from the same preparation in the Ctrl solution for 5 or 20 min and then analyzed the material from both time points on the same western blot. As above, we first normalized PLC-γ1.pY783 or p-Erk1/2 immunoreactivity to the corresponding total-protein immunoreactivity on reprobed blots. We then normalized both 5-min and 20-min values to the 5-min value, and performed a log_2_ transformation. We find that the time dependence of PLC-γ1.pY783 is not remarkable. However, p-Erk1 has a tendency to decline, and p-Erk2 significantly declines.

## Discussion

### General issues

Previous work on PTs showed that raising [CO_2_]_BL_ or lowering [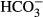]_BL_ under OOE conditions causes *J*hco_3_ to fall acutely. Moreover, the [CO_2_]_BL_-dependent effects on *J*hco_3_ require a kinase (presumably ErbB1 and/or ErbB2) that is blocked by PD168393 and BPIQ-1 (Zhou et al. 2006a), the secretion of ANG II into the PT lumen (Zhou and Boron 2008), and the binding of ANG II to apical AT_1A_ receptors (Zhou et al. 2007). These observations raise the question of whether the transduction of signals between changes in [CO_2_]_BL_ and responses of PT acid-base transporters may involve ErbB1/2 as well as enzymes that are downstream of ErbB1/2 (e.g., PLC-γ1, Erk1/2) (Yarden and Sliwkowski 2001; Normanno et al. 2006; Roskoski 2014) and AT_1A_ (Lea et al. 2002). Indeed, an earlier study showed that MAc, RAc, MAlk, RAlk, and cRAc each produce characteristic pY changes in ErbB1/2 that evolve from 5 to 20 min of treatment (Skelton and Boron 2013). In the present work, we find that the same acid-base disturbances have characteristic effects on the phosphorylation status of PLC-γ1 or Erk1/2 over the same time range.

#### Sensors

A key question—not addressed in the present study—concerns the identity of the sensor(s) for basolateral CO_2_ and 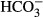. In principle, ErbB1 or ErbB2 could themselves be sensors. However, if they are sensors for CO_2_ or 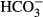, they cannot be the only sensors, because in the present study we show that some responses proceed even in the presence of PD168393.

#### Phosphorylation of Erk1/2

Note that when we discuss p-Erk1 and p-Erk2, we refer specifically to pT202 and pY204 (human numbering) within a conserved TEY activation loop motif of Erk1, and the homologous pT185 and pY187 residues (human numbering) of Erk2. The dual-specificity kinases MEK1/2—downstream of ErbB1/2 but upstream of Erk1/2—phosphorylate both the threonine and tyrosine residues in the TEY motif. Indeed, the dual pT+pY phosphorylation is required for maximal Erk activity (Payne et al. 1991; Canagarajah et al. 1997). The polyclonal phospho-specific antibody that we used in the present study detects pT and pY, individually or together. Other consensus Erk1/2 phosphorylation sites^4^ could also contribute to the role of Erk1 and Erk2 in acid-base signaling.

#### Statistical analyses

Our statistical analyses in the present study use the same conservative approach as for a similar study on the effects of acid-base disturbances on the phosphorylation state of tyrosine residues in ErbB1 and ErbB2 (Skelton and Boron 2013). In the figures, a # indicates data for which *P* < 0.05, compared to zero, in one-tailed, paired *t*-test; in the text, we state that the parameter “tends” to rise/fall. Inasmuch as we make several parallel comparisons in each figure panel, this t-test may not sufficiently stringent. On the other hand, a * in the figures indicates statistical significance even after the very conservative Bonferroni correction; in the text, we refer to the change as “significant.” Of course, a change may be real even though the data set lacks the statistical power to pass a statistical test, especially a test that follows a very stringent Bonferroni correction.

#### Native tissue

We performed all of our studies on native tissue, as opposed to cultured cells under highly controlled conditions, and heterologously expressing artificially high levels of one or more proteins. Although our conservative approach has the advantage that the observed changes are more likely to be physiological, it has the disadvantage that changes tend to be small. One reason for small changes could be that a particular protein could be in different physiological states and various locations within PT cells. The combination of stringent statistical analyses and small overall changes in phosphorylation can combine to make it appear that effects are less robust than they may actually be within a certain microenvironment of a native PT cell. Knowing that a particular disturbance (e.g., RAc) has a strong effect (e.g., decreased PLCγ-1.pY783 at 20 min) in native tissue, one might now approach the problem in a model system with overexpressed heterologous proteins.

### Comparison of phosphorylations at 5 and 20 min in nominally CO_2_/HCO^−^_3_-free solutions

**Figure 10:**
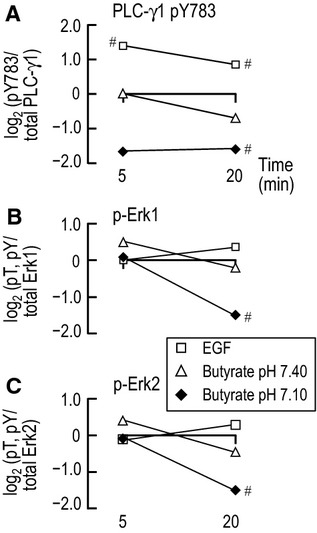
Time-dependent changes of PLC-γ1 and Erk1/2 phosphorylation in nominally CO_2_/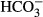-free solutions. Here we replot, as a function of time, the log_2_ transformed data for PLC-γ1.pY783 (Figs. 3A, 4A) and p-Erk1/2 (Figs.7A, 8A) and group by enzyme: (A) PLC-γ1.pY783, (B) p-Erk1, and (C) p-Erk2. Recall that these data are all normalized to the HEPES treatment. ^#^Denotes a bar for which *P* < 0.05 (compared to 0, one-tailed, paired *t*-test).

Figure10A summarizes the time course of PLC-γ1.pY783 in nominally CO_2_/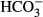-free solutions by synthesizing data from Figure3A (at 5 min) and Figure4A (at 20 min). EGF produces a tendency for PLC-γ1.pY783 to increase at 5 and 20 min. The effect of butyrate/pH 7.1 to reduce PLC-γ1.pY783 barely misses reaching significance at the 0.05 level in a paired t-test at 5 min, but does reach this level at 20 min. Thus, it is likely that that butyrate/pH 7.1 causes a sustained decrease in PLC-γ1.pY783. The effect of butyrate/pH 7.4 is unremarkable.

Figure10B summarizes the time course of changes in p-Erk1 from Figures7A, 8A, 10C does the same for p-Erk2. None of the treatments is remarkable for either p-Erk1 or p-Erk2 at either 5 or 20 min except for Butyrate/pH 7.1, which tends to decrease both p-Erk1 (Fig.10B) and p-Erk2 (Fig.10C) at 20 min. Indeed, our previous work on ErbB1/2 phosphorylation showed that butyrate is more likely to have effects, and stronger effects, at pH 7.1 versus 7.4 (Skelton and Boron 2013). It is perhaps surprising that EGF does not increase p-Erk1 (Fig.10B) or p-Erk2 (Fig.10C) at 5 or 20 min. However, note that the ErbB inhibitor PD168393 is ineffective in lowering p-Erk1/2 levels in the absence of CO_2_/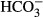 (see HEPES bars in Figs.7C, 8C). Thus, it may be that—at least in PT suspensions—ErbB1/2 cannot effectively stimulate Erk1/2 (as opposed to PLC-γ1) in the nominal absence of CO_2_/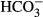.

### Comparison of PLC-γ1 and Erk1/2 phosphorylation after 5- and 20-min treatments in CO_2_/HCO^−^_3_-containing solutions that simulate classic acid-base disturbances

**Figure 11:**
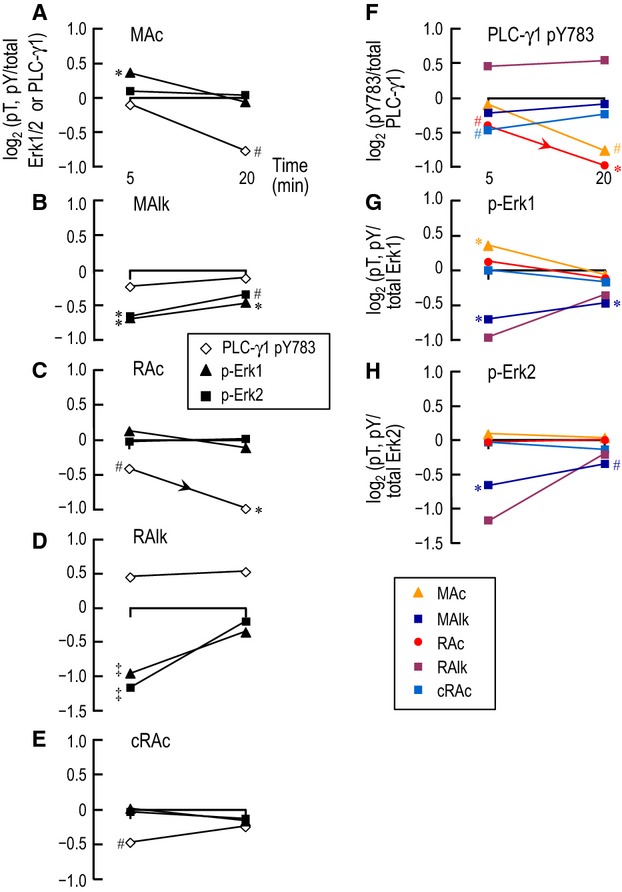
Time-dependent changes of PLC-γ1 and Erk1/2 phosphorylation in CO_2_/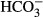 solutions during acid-base disturbances. Here we replot, as a function of time, the log_2_ transformed data for PLC-γ1.pY783 (Figs. 3B, 4B) and p-Erk1/2 (Figs. 7B, 8B) and group by either acid-base disturbance (A–E) or enzyme (F–H) Recall that these data are all normalized to the Ctrl treatment. ^#^Denotes a symbol for which *P* < 0.05 (compared to 0, one-tailed, paired t-test). * Denotes a symbol that is significantly different from 0, even after the very conservative Bonferroni correction (*P* < 0.05 divided by the number of treatment solutions considered in Figs.3B, 4B, 7B, 8B). ^‡^Denotes a symbol that differs significantly from RAc at 5 min in a one-tailed paired *t*-test. The downward-sloping arrowheads indicate a statistically significant difference between the corresponding values at 5 and 20 min in a one-tailed paired *t*-test.

**Figure 12:**
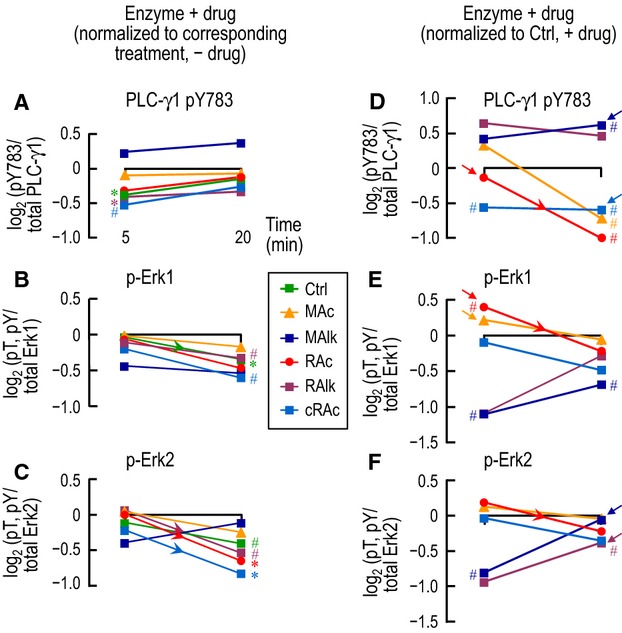
Time-dependent changes of PLC-γ1 and Erk1/2 phosphorylation in PD168393-containing CO_2_/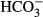 solutions during acid-base disturbances. (A–C) Here we replot, as a function of time, the log_2_ transformed data for PLC-γ1.pY783 (Figs.3C, 4C) and p-Erk1/2 (Figs.7C, 8C) and group by enzyme. Recall that these data are all normalized to the equivalent treatments without PD168393. (D–F) Here we replot, as a function of time, the log_2_ transformed data for PLC-γ1.pY783 (Figs.3D, 4D) and p-Erk1/2 (Figs.7D, 8D) and group by enzyme. Recall that these data are all normalized to the Ctrl treatment with PD168393. ^#^Denotes a symbol for which *P* < 0.05 (compared to 0, one-tailed, paired t-test). *Denotes a symbol that is significantly different from 0, even after the very conservative Bonferroni correction (*P* < 0.05 divided by the number of treatment solutions considered in Figures3C or D, 4 C or D, 7 C or D, Fig.8 C or D). The downward-sloping arrowheads indicate a statistically significant difference between the corresponding values at 5 and 20 min in a one-tailed paired *t*-test. The arrows in panels D–F denote a symbol for which the statistical outcome in the present figure (presence of drug) differs from the statistical outcome for the same symbol in Fig.11F–H (without drug).

In Figure11A–E, we show the phosphorylation time course of the three enzymes, grouped by acid-base condition. In each panel, we synthesized data from Figures12B, 3B, 6B, 7B, comparing the levels of phosphorylation at 5 min and at 20 min in CO_2_/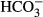-containing solutions, each data point normalized to phosphorylation levels in Ctrl at 5 min or at 20 min, as appropriate.

#### MAc

PLC-γ1.pY783, relative to Ctrl, shows a tendency to fall at 20 min (Fig.11A). Although p-Erk1 and p-Erk2 generally follow one another closely, both at 5 and 20 min, their behavior with MAc is an exception. Here, p-Erk1, but not p-Erk2 rises relative to Ctrl at 5 min (but not at 20 min).

#### MAlk

PLC-γ1.pY783 appears not to be under the strong influence of MAlk (Fig.11B). However, p-Erk1 and p-Erk2 fall similarly with MAlk, both at 5 and 20 min. Note that, for all three enzymes, the phosphorylation pattern with MAlk (Fig.11B) is distinct from that with MAc (Fig.11A).

#### RAc

PLC-γ1.pY783 falls at 5 min and more so at 20 min (Fig.11C). Note that the pY783 responses at 20 min are similar for the two acidoses, MAc (Fig.11A) and RAc (Fig.11C), and that both differ from the responses to the two alkaloses (Fig.11B, D) and the isohydric condition (Fig.11E). RAc does not appear to strongly influence p-Erk1 or p-Erk1/2.

#### RAlk

PLC-γ1.pY783 appears not to be under the strong influence of RAlk (Fig.11D). This is the same conclusion we reached above for MAlk. Although, by themselves, the data for p-Erk1 and p-Erk2 do not have the statistical power to reach significance relative to Ctrl at 5 min, the pairwise comparison between RAlk and RAc at 5 min (Fig.7) does reveal a difference. Thus, the two alkaloses (MAlk vs. RAlk) may produce similar effects at 5 min, but differ at 20 min for the three enzymes in the present study.

#### cRAc

PLC-γ1.pY783 tends to fall at 5 min but not at 20 min (Fig.11E). This 5-min response is similar to that of the other high-CO_2_ condition (RAc, Fig.11C), although the cRAc and RAc responses are distinct at 20 min. p-Erk1 and p-Erk2 appear not to be influenced by cRAc (Fig.11E), reminiscent of the lack-of-response to RAc (Fig.11C).

In Figure11F–H, we view the time analysis from a different perspective, grouping all acid-base disturbances together for each of the three enzymes. Although each enzyme has a characteristic pattern of responses to the group of acid-base disturbances, no single enzyme (PLC-γ1, Erk1, Erk2) can distinguish among all five acid-base disturbances. In our previous study (Skelton and Boron 2013), we similarly found that no single one of the four pY sites on ErbB1 or the dual pY site on ErbB2 can distinguish among the disturbances. In other words, of the eight nonredundant phosphorylation sites on five enzymes that we have examined in the two studies, no one site by itself can distinguish among the five acid-base disturbances.

Although no one phosphorylation site can distinguish among the five acid-base disturbances, a combination of sites can. For example, Figure11A–E in the present study shows that each of the five disturbances produces a distinct pattern of changes even within the limited phosphorylation space of PLC-γ1.pY783, p-Erk1, and p-Erk2. Similarly, our earlier study showed that each of the five disturbances produces a distinct pattern of changes within the phosphorylation space consisting of four pY sites on ErbB1 and the dual site on ErbB2 (see fig. 13A–E in ref. Lea et al. 2002). Although not shown, a consolidation of the data from the present study (1 site on PLC-γ1 plus compound sites on each of Erk1 and Erk2) and the previous one (4 pY sites on ErbB1 plus a dual site on ErbB2) would reveal an even stronger set of characteristics that distinguish each of the five acid-base disturbances from the other four. Data consolidation from the two studies also would reveal strong sets of characteristics shared among disturbances with common changes in acid-base parameters (e.g., those having in common a decrease in pH_o_ or an increase in [CO_2_]_o_). Thus, the PT cell—even in a phosphorylation space confined to eight nonredundant sites on five proteins—has available to it a wealth of time-dependent signaling (i.e., phosphorylation status) information.

Although, in this and the previous study (Skelton and Boron 2013), we have obtained phosphorylation data only at 5 and 20 min, it is possible that the changes in phosphorylation begin before 5 min, further evolve between our 5- and 20-min time points, and continue to evolve even after the 20-min point. Moreover, each acid-base disturbance likely triggers changes in the phosphorylation status of many more proteins than those we examined in the present and previous studies (Skelton and Boron 2013). Thus, each acid-base disturbance would produce a characteristic pattern of phosphorylation status, evolving in time for each of a collection of many target proteins, often (as is the case for ErbB1/2) at multiple potential phosphorylation sites for a single protein. This large and unique collection of phosphorylation time courses is the fingerprint that, at least in part, instructs the cell how to respond, over time, to each acid-base disturbance.

### Effect of PD168393 on the responses of PLC-γ1.pY783 and p-Erk1/2

An analysis of the effects of PD168393 on the phosphorylation patterns provides insight into the involvement of signaling from PD168393-sensitive kinases, presumably ErbB1/2. Figure12A–C shows a time course synthesized from PD168393-treated samples in Figures12B, 3B, 6B and 7B, where we normalized the data to the corresponding treatment without drug. Thus, these plots show how PD168393 (i.e., blockade of ErbB1/2) affects phosphorylation status during any one acid-base disturbance. PLC-γ1.pY783 has noteworthy PD168393 sensitivity only at 5 min and only for Ctrl, RAlk, and cRAc (Fig.12A). Neither p-Erk1 nor p-Erk2 exhibit noteworthy PD168393 sensitivity at 5 min under any condition, but both develop sensitivity for several conditions at 20 min (Fig.12B–C). Thus, signaling via ErbB1/2 to PLC-γ1, Erk1, and Erk2 is dynamic (i.e., time dependent) and depends on the acid-base disturbance or lack thereof.

Figure12D–F shows a time course synthesized from PD168393 data from Figures12D, 3D, 6D, 7D, where we normalized the data to our Ctrl + PD168393. Thus, these plots show how each acid-base disturbance affects phosphorylation status in the presence of PD168393 (i.e., absence of ErbB1/2). We see that, even in the absence of ErbB1/2 activity, the acid-base disturbances affect the time course of phosphorylation for each of the three proteins. Thus, acid-base disturbances do no signal exclusively through ErbB1/2. Comparing the timelines in Figure12D–F (presence of PD168393) with those in Figure11F–H (absence of PD168393) reveals several differences—indicated by arrows—in the outcomes of the statistical analyses. We will consider these differences in the following five paragraphs.

Table2 summarizes—for each enzyme—the change in phosphorylation (↓, no change, ↑) in response to each of the five acid-base disturbances in the absence of PD168393 (taken from Fig.11F–H) and in the presence of the drug (taken from Fig.12D–F). A blank cell in Table2 means that none of the three phosphorylation levels changes in a noteworthy fashion, either without or with the drug, in the present study. Thus, RAlk at 5 min does not have noteworthy effects (with data compared to control) for any of the three enzymes. Nevertheless, it is possible that—if our data had greater statistical power—additional effects may have emerged.

**Table 2:** Patterns of phosphorylation responses, ±PD168393^1^

	Effect of various acid-base disturbances on phosphorylation, ±drug		RAlk		Mac		MAlk		RAc		cRAc	
	−PD168393	+PD168393	5 min	20 min	5 min	20 min	5 min	20 min	5 min	20 min	5 min	20 min
1	↓	↓				pY783 (Fig.4)	p-Erk1 p-Erk2 (Fig.7)	p-Erk1 (Fig.8)		pY783 (Fig.4)	pY783 (Fig.3)	
2	↓	No Δ						p-Erk2 (Fig.8)	pY783 (Fig.3)			
3	↑	No Δ			p-Erk1 (Fig.7)							
4	No Δ	↓		p-Erk2 (Fig.8)								pY783 (Fig.4)
5	No Δ	↑						pY783 (Fig.4)	p-Erk1 (Fig.7)			

1 Responses −PD168393 come from the B panels of Figs.3, 4, 7, 8. Responses +PD168393 come from the D panels of Figs.3, 4, 7, 8. Not shown are responses with the pattern “No Δ, No Δ”. We found no examples of the patters ↓↑, ↑↑, ↑↓.

Table2/row 1 (downward arrows in first two columns, ↓/↓) shows that MAc × 20 min lowers PLC-γ1.pY783 without or with the drug. In other words, the ability of MAc to lower pY783 does not depend on a PD168393-sensitive kinase. Four other situations produce the same ↓/↓ pattern in one or more enzymes—that is, PD168393-insensitive effects. Thus, some molecule(s) independent of ErbB1/2 must be able to sense CO_2_ and 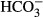 and initiate the transduction of signals that shift kinase/phosphatase balance to decrease PLC-γ1.pY783 or p-Erk1 or p-Erk2.

On the other hand, PD168393-sensitive kinase activity must be important because—as summarized in Table2/row 2 (↓/No change) and Table2/row 3 (↑/No change)—the drug can eliminate certain responses to MAc, MAlk, and RAc.

Rows 4–5 of Table2 reveal that RAlk, MAlk, RAc, and cRAc can produce effects too small for us to detect in the absence of PD168393, but produce noteworthy changes in phosphorylation in the presence of drug. These results imply a rough balance—in the absence of PD168393—between parallel response pathways involving drug-sensitive and drug-insensitive kinases/phosphatases.

Note that we did not observe responses in the combinations ↓/↑, ↑/↑, or ↑/↓.

### Physiological relevance

Our systematic analysis of how the four fundamental acid-base disturbances, as well as fully compensated RAc (i.e., cRAc)—which could also be called fully compensated MAlk—affect three key enzymes reveals several noteworthy responses. Some of these correlate in intriguing ways with the previous work of others on signal-transduction processes affecting acid-base transport.

Although ErbB activation can lead to the phosphorylation of at least four PLC-γ1 tyrosine residues (Kim et al. 1990), it is phosphorylation at Y783 that is necessary for activating PLC-γ1 lipase activity (Kim et al. 1991; Poulin et al. 2005). Moreover, others have shown that activated PLC-γ1 translocates to apical membrane of ileal enterocytes, where it directly binds to the C terminus of NHE3, and serves as a docking site for c-Src—part of the Ca^2+^-induced, Src-dependent inhibition of NHE (Khurana et al. 1996; Zachos et al. 2009, 2013). Thus, we find it intriguing that MAc and RAc at 20 min reduce PLC-γ1.pY783, independent of PD168393-sensitive kinases (see Fig.4B, Table2/row 1). It is possible that MAc and RAc, by shifting the kinase/phosphatase balance and thereby lowering PLC-γ1.pY783, disinhibit NHE3 and raises *J*hco_3_—the appropriate response to acidosis.

Others have implicated Erk1/2 as key players in the regulation of acid-base transport by the PT. For example, Tsuganezawa et al. (2002) found that MAc, induced by a 15-min NH_4_Cl gavage in rats, increases Erk1/2 activity in the rat renal cortex. Moreover, Li et al. (2008) found that low-dose ANG II increases in p-Erk1/2 in mouse renal cortex, and that a MEK inhibitor blocks the ability of low-dose ANG II to stimulate NBCe1-A activity in mouse PTs. Our laboratory has shown that both PD168393-sensitive kinases (Zhou et al. 2006a) and AT_1A_ signaling at the apical membrane (Zhou and Boron 2008) are necessary for increases in [CO_2_]_BL_ to increase *J*hco_3_ in PTs, and it is reasonable to hypothesize that the ability of decreases in [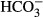]_BL_ to increase *J*hco_3_ has similar requirements. In the present study, we find that MAc at 5 min—the only acid-base disturbance that increases any phosphorylation in the absence of PD168393—increases p-Erk1, and that this effect is blocked by PD168393 (Fig.7, Table2/row 3). Conversely, MAlk at 20 min lowers p-Erk2 (Fig.8, Table2/row 2). Viewed just in the context of our data, one might postulate that signaling proceeds as follows: acidosis → ErbB1/2 → MEK1/2 → ERK → apical AT_1A_ → → NBCe1-A. However, the data of Li et al. suggest an alternative sequence: acidosis → ErbB1/2 → → apical AT_1A_ → → MEK1/2 → ERK → → NBCe1-A.

Also consistent with the above trends are—in the absence of PD168393—MAlk at 5 min, which decreases p-Erk1/2 (Fig.7, Table2/row 1), and MAlk at 20 min, which decreases p-Erk1 (Fig.8, Table2/row 1). However, in all three of these cases, the decreased phosphorylation persists in the presence of PD168393. Thus, MAlk must be able to decrease ErbB-insensitive kinase activity or increase phosphatase activity independent of ErbB signaling.

## Conclusion

Subjecting rabbit PTs to acid-base disturbances causes characteristic alterations in the phosphorylation status of PLC-γ1 (pY783), Erk1 (pT202/pY204), and Erk2 (pT185/pY187), consistent with a role in the transduction of acid-base signals in the PT.

## Footnotes

1 *Abbreviations used*: cRAc, fully compensated respiratory acidosis; MAc, metabolic acidosis; MAlk, metabolic alkalosis; RAc, respiratory acidosis; RAlk, respiratory alkalosis.

2 Other options exist. For example, MAlk could stimulate a PD168393-sensitive kinase that, in turn, stimulates a phosphatase that dephosphorylates PLC-γ1.pY783.

3 We observed several such examples in our previous work on ErbB1 and ErbB2 (Skelton and Boron 2013). For example, MAlk tends to increase in ErbB1-pY845 at 5 and 20 min (see Fig.7B, 8B in ref. Lea et al. 2002), and cRAc increases ErbB1-pY845 at 20 min (see Fig.7B, 8B in ref. Lea et al. 2002)

4 See the Cell signaling database http://www.phosphosite.org/homeAction.do for other potential sites on Erk1 and Erk2
